# Biomechanical Comparison of Six Different Root-Analog Implants and the Conventional Morse Taper Implant by Finite Element Analysis

**DOI:** 10.3389/fgene.2022.915679

**Published:** 2022-06-13

**Authors:** Jia-Qing Wang, Yuan Zhang, Min Pang, Yue-Qiu Wang, Jun Yuan, Hui Peng, Wen Zhang, Lu Dai, Hong-Wei Li

**Affiliations:** ^1^ Department of Oral and Maxillofacial Surgery, The Affiliated Stomatological Hospital of Nanjing Medical University, Nanjing, China; ^2^ Jiangsu Province Key Laboratory of Oral Diseases, Nanjing, China; ^3^ Jiangsu Province Engineering Research Center of Stomatological Translational Medicine, Nanjing, China; ^4^ The Fourth Outpatient Department, The Affiliated Stomatological Hospital of Nanjing Medical University, Nanjing, China

**Keywords:** finite element analysis, root-analog implant, tapered implant, biomechanics, stress

## Abstract

Taper implants differ greatly from anatomical teeth in shape. In this study, seven three-dimensional finite element models were established, including a conventional taper implant and six root-analog implants with different root numbers and shapes. Vertical, horizontal, and oblique instantaneous loads of 100 N were applied to the models to obtain stress distribution in the implant, mucosa, cortical bone, and cancellous bone. ANSYS was used to perform the analysis under hypothetical experimental conditions. We find the stresses in all the implants and surrounding tissues varied by loading direction, the sequence of stress magnitude is vertical load, oblique load, and then horizontal load. The maximum stress values in root-analog implants were significantly less than in the taper implant. Moreover, stress distribution in the former was equalized contrary to the concentrated stress in the latter. Root-analog implants with different root geometry also revealed a pattern: stresses in multiple-root implant models were lower than those in single-root implants under the same load. The implant with a long and rounded root distributed the stress more uniformly, and it was mainly concentrated on the implant itself and cancellous bone. However, the opposite effect was observed in the short implant on mucosa and cortical bone. The root geometry of anatomical teeth can modify their functions. A uniform-shaped implant can hardly meet their functional requirements. Thus, the root-analog implant could be a possible solution.

## Introduction

Human teeth developed into heteromorphic thecodont dentition millions of years ago (David Polly, 2012; Evans et al., 2016; Ortiz et al., 2018). Based on the functions, teeth are divided into various groups anterior to the oral cavity. Incisors are involved in cutting food, assisting pronunciation, and affecting facial aesthetics. Being adjacent to incisors, canines penetrate and tear food and contribute to facial appearance. Molars and premolars grind food into smaller pieces. Based on the teeth measurements in Chinese people, the geometry differs greatly in different teeth types. Every tooth has specific measurements for its crown, neck, and root (Wang, 1959). For example, the canine root is much longer than any other tooth. In contrast, molars usually have two or three roots. These differences suggest that the teeth geometry is generally compatible with their specific functions (Torices et al., 2018; Krings et al., 2020). Thus, the loss of any type of tooth leads to different functional defects.

Oral Implantology was acknowledged as the optimal method of repairing dentition defects (Buser et al., 2017). However, dental implants are currently taper or cylindrical shaped, which are far different from anatomical teeth in terms of the root geometry (Pirker and Kocher, 2008). For example, a maxillary first molar has three roots to withstand masticatory force. However, all this force falls on one implant after the tooth is lost, and consequently, stress concentration and other biomechanical incompatibilities may arise (Gotfredsen et al., 2002; Daubert et al., 2015; Bing et al., 2020). Based on these results, we hypothesized that if the implant’s geometry is root-analog and it is composed of titanium, then stress distribution on the implant will improve or not.

To compare the biomechanical properties of titanium root-analog implants and the taper implant, and to understand the relationship between root geometry and their corresponding function, it seems essential to analyze the stress distribution in implants and their surrounding tissues under certain masticatory force. Due to the difficulty of human experimentation and the complex structure of teeth, it is necessary to divide the complex structure, including tooth and surrounding tissues, into simple discrete domains with finite element analysis (FEA) (He et al., 2017; Oliveira et al., 2020).

FEA refers to the method of using a mathematical approximation to simulate a physical system. It is a powerful tool for analyzing the mechanics of irregularly shaped objects. Since 1976, when Weinstein et al. (1976) applied FEA first time to oral implantology, this technology had become an important method for analyzing stress distribution in dental implants, abutment, and crowns (Santos et al., 2009; Dos Santos Marsico et al., 2017; Kang et al., 2020). FEA showed better stress distribution than the conventional taper implant for root-analog implant. In this case, only the single-root implant was involved (He et al., 2017; Dantas et al., 2020). However, FEA of root-analog implants with double roots and three roots remain unexplored.

Titanium is a widely used dental implant material, as it is the most biocompatible metal implant material (Buser et al., 2017). Therefore, in the present study, six root-analog implants from different tooth types were selected for three-dimensional titanium FEA to compare their stress distribution with the taper implant. The relationship between the teeth geometry and root-analog implants, including root length, cross-sectional geometry, number of roots, and function, was clarified.

## Materials and Methods

### The Establishment of Root-Analog and Taper Implants Finite Element Analysis Models

**FIGURE 1:**
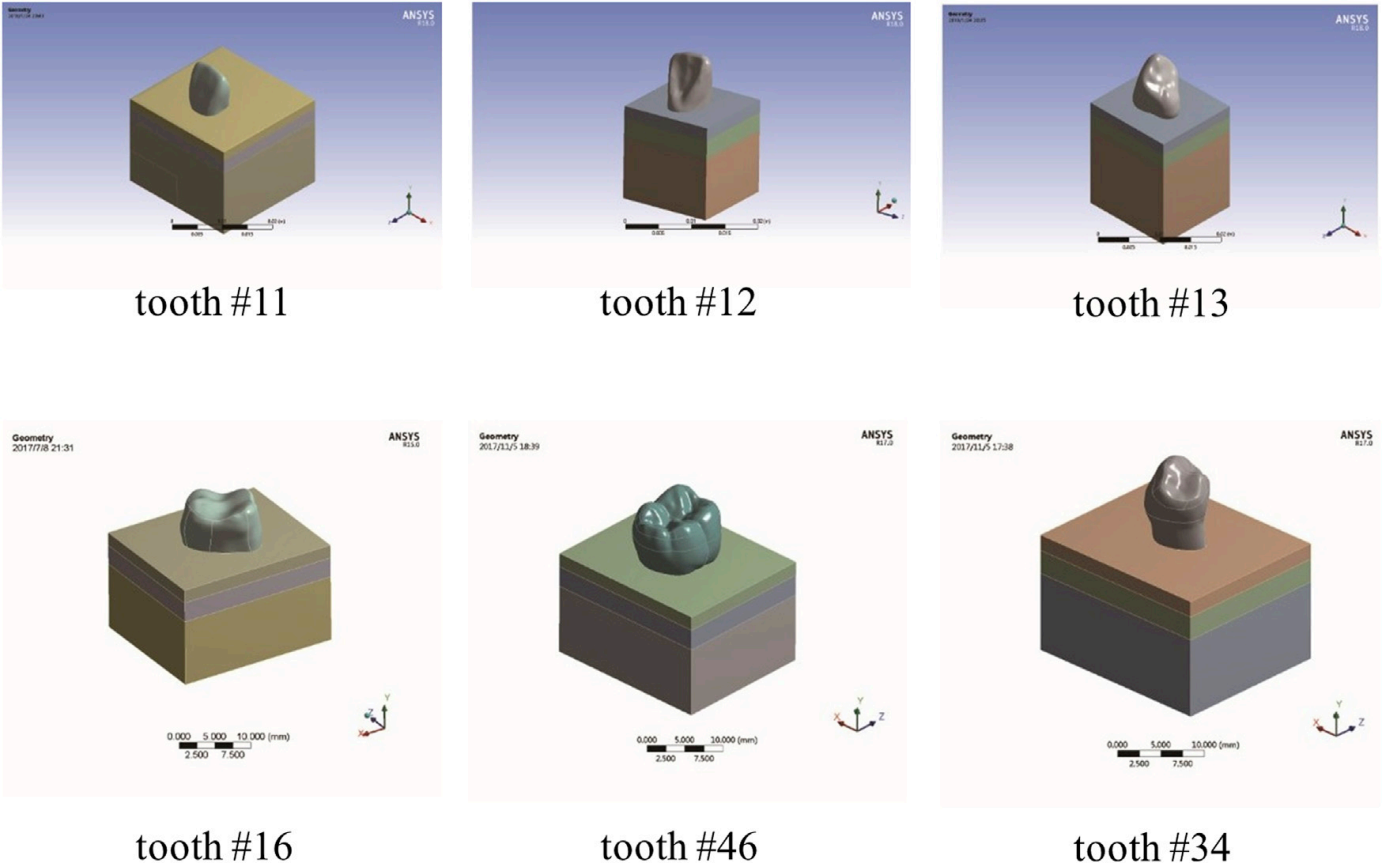
FEA models of RAIs and surrounding tissues.

Standard anatomical teeth model of maxillary central incisor, maxillary lateral incisor, maxillary cuspid, maxillary first molar, mandibular first molar, and mandibular first premolar was scanned using a microcomputed tomography (micro-CT) scanner (Skyscan1172, Micro Photonics Inc., Allentown, PA). Next, transverse sections were generated and processed using Mimics v21.0 (Materialise, Leuven, Belgium) (He et al., 2017; Kang et al., 2020; Oliveira et al., 2020). Then, we imported the model files into ANSYS workbench18.0 (ANSYS, Inc., Canonsburg, PA, United States) and established the finite element models consisting of root-analog implants and their surrounding tissues, including mucosa, cortical bone, and cancellous bone (Figure 1). A morse taper implant system was modeled using Cinema 4D (Maxon Computer, Friedrichsdorf, Germany). The geometrical characteristics are a length of 18 mm, an external diameter of 4.2 mm, and a healing abutment (4.5*8.0 mm), which were based on the commercially available components (ADIN^©^ Dental Implant Systems, Ltd., Afula, IL). Finally, the taper implant was assembled with the surrounding tissues in ANSYS.

### Simulation Condition

The experimental conditions were as follows: 1) All tissues and materials were isotropic elastic materials; 2) The root-analog and taper dental implants were 100% bonded to the alveolar bone; 3) The thickness of mucosa was 2 mm, and the cortical bone was 3 mm. The whole root of each implant was located within the cancellous bone, and mucosa-air junction plane was located in the cervix of the implants; and 4) The implant material in the models was titanium. The mechanical properties (Dantas et al., 2020) of the model-related materials are presented in Table 1. The implants and their crowns or abutment were integrated because the root-analog implants are usually one-piece (Pirker et al., 2011; Saeidi Pour et al., 2019).

**TABLE 1:** Elastic modulus and Poisson ratio of different materials.

Material	Elastic modulus (GPa)	Poisson ratio (μ)
Cortical bone	13.7	0.30
Cancellous bone	1.85	0.30
Mucosa	0.003	0.45
Titanium	110	0.35

### The Protocol of Finite Element Analysis

ANSYS workbench was used to load the FEA models with hypothetical experimental conditions (Dantas et al., 2020; Kang et al., 2020). Based on our experimental design, we applied vertical, horizontal, and oblique instantaneous loads of 100N on the models to simulate the masticatory force on teeth daily. The vertical load was applied on the center of the occlusal surface, its direction was consistent with the long axis of the implants. The horizontal load was applied on the central buccal surface, and its direction was perpendicular to the long axis of the implants. The oblique loading point was the same as the horizontal load, but its direction was 45° with the long axis of the implants. The stress distribution, maximum and minimum stress values in the root-analog implants, the taper implant, mucosa, cortical bone, and cancellous bone were obtained under all loads from different directions.

### Results Analysis

Based on the root morphology, we divided the root-analog implants into four groups for comparison: 1) Group A, based on the number of roots: maxillary first molar with three roots, a mandibular first molar with two roots, and maxillary central incisor with a single root; 2) Group B, based on the length of the root: maxillary central incisor, maxillary lateral incisor, and maxillary cuspid; 3) Group C, based on the length–width ratio of the root cross-section: maxillary central incisor was a round root model and mandibular first premolar was a flat root model; and 4) Group D, the taper implant.

## Results

**FIGURE 2:**
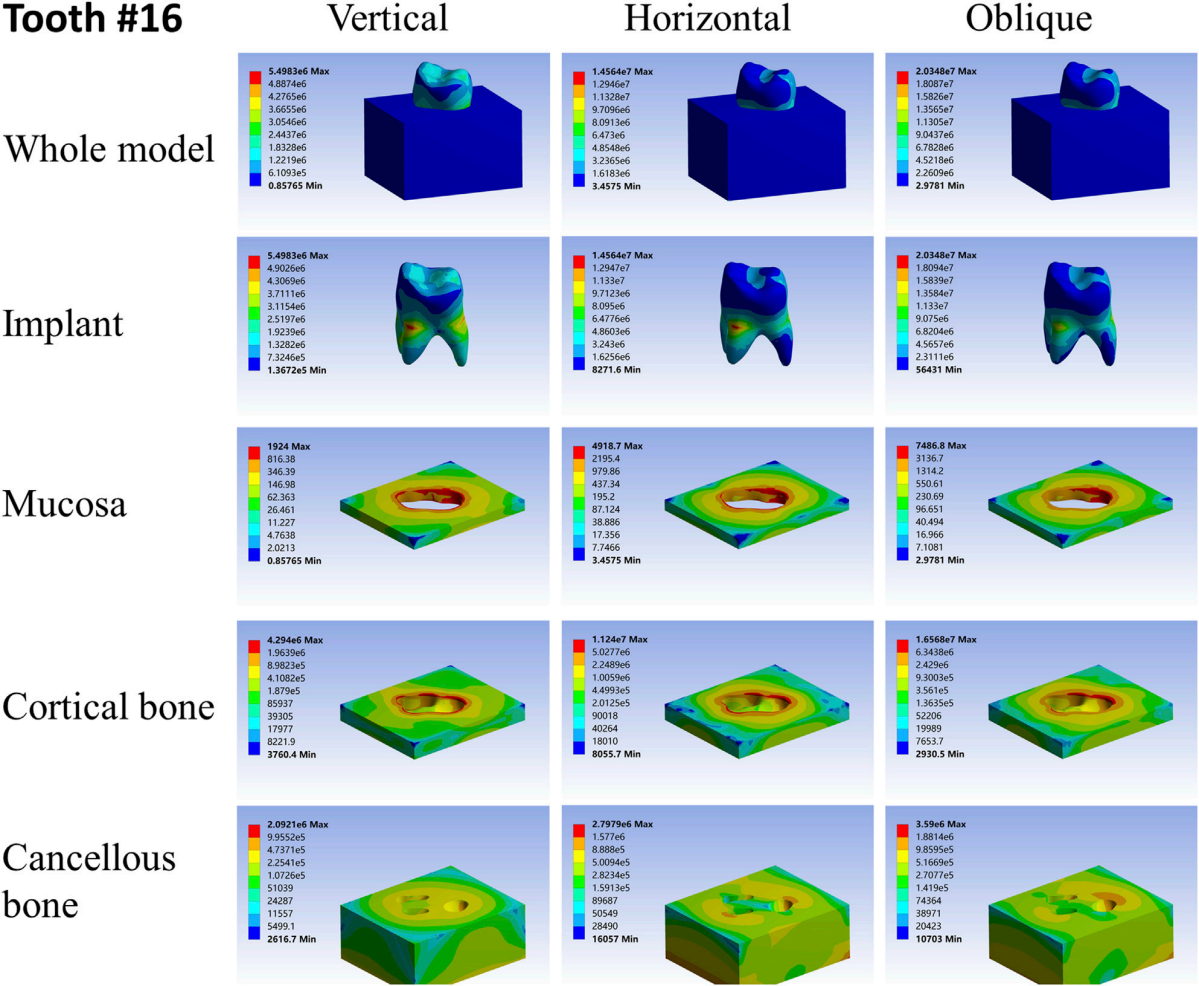
Stress distribution of 26 under 100-N load.

**FIGURE 3:**
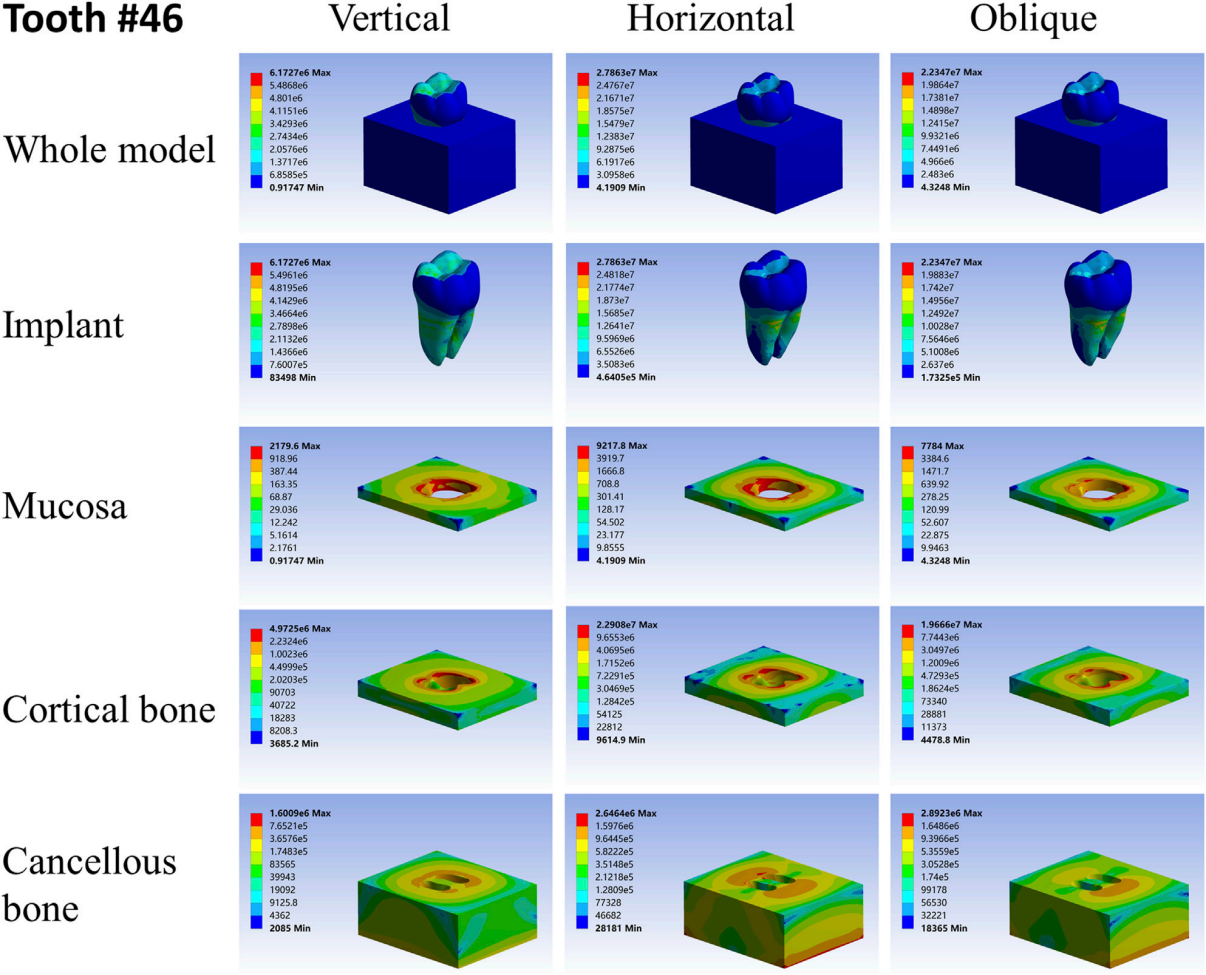
Stress distribution of 46 under 100-N load.

**FIGURE 4:**
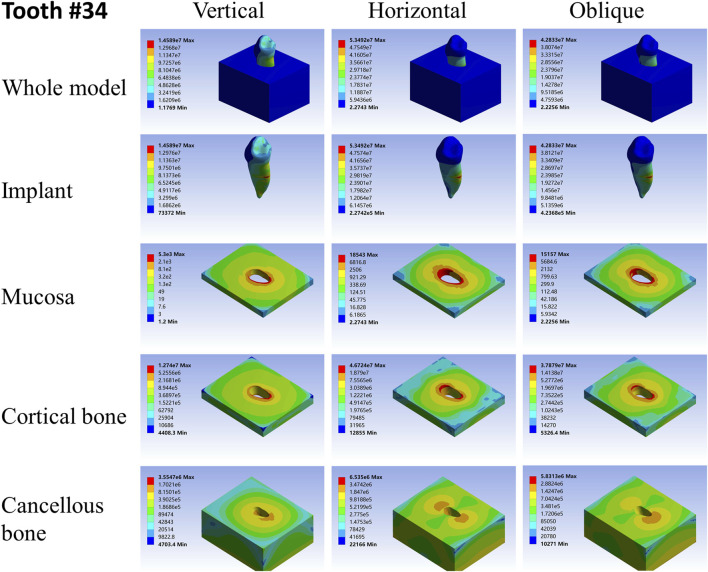
Stress distribution of 34 under 100-N load.

**FIGURE 5:**
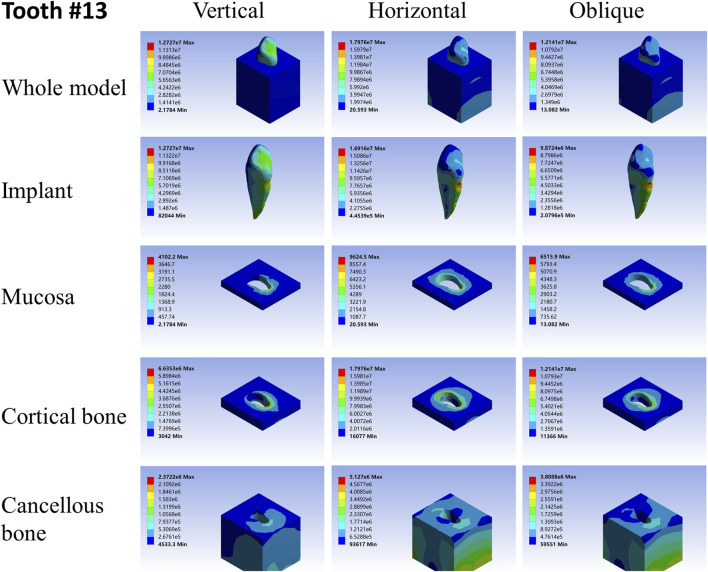
Stress distribution of 13 under 100-N load.

**FIGURE 6:**
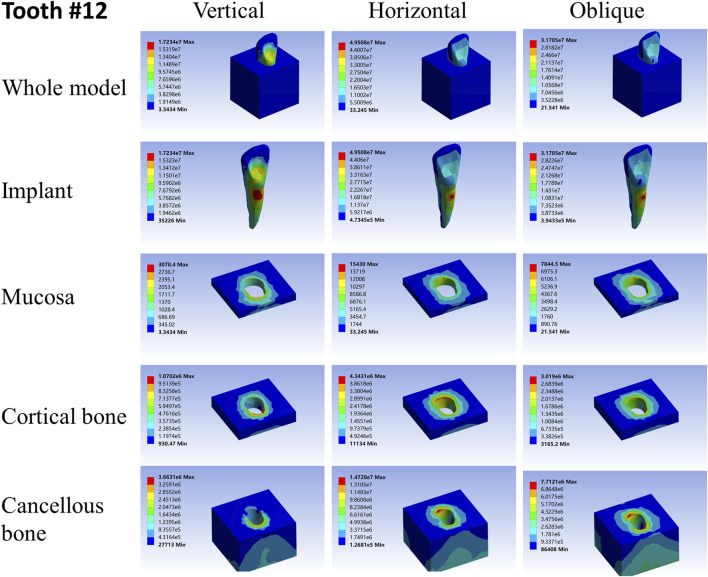
Stress distribution of 12 under 100-N load.

**FIGURE 7:**
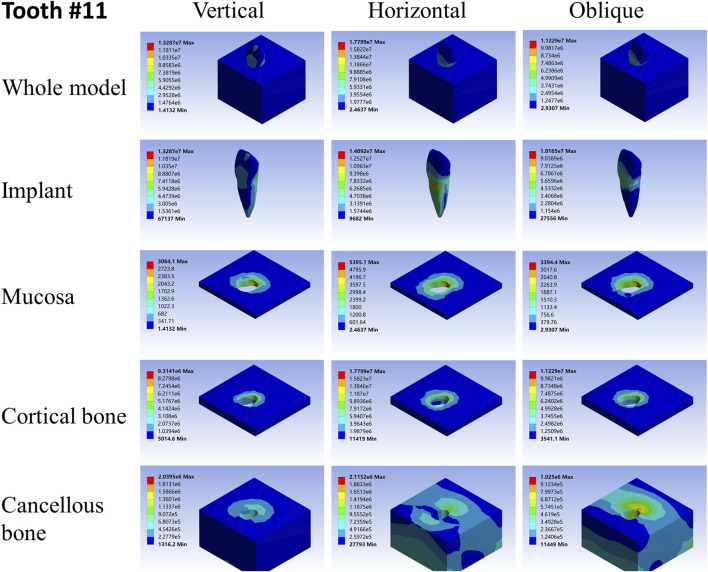
Stress distribution of 11 under 100-N load.

**FIGURE 8:**
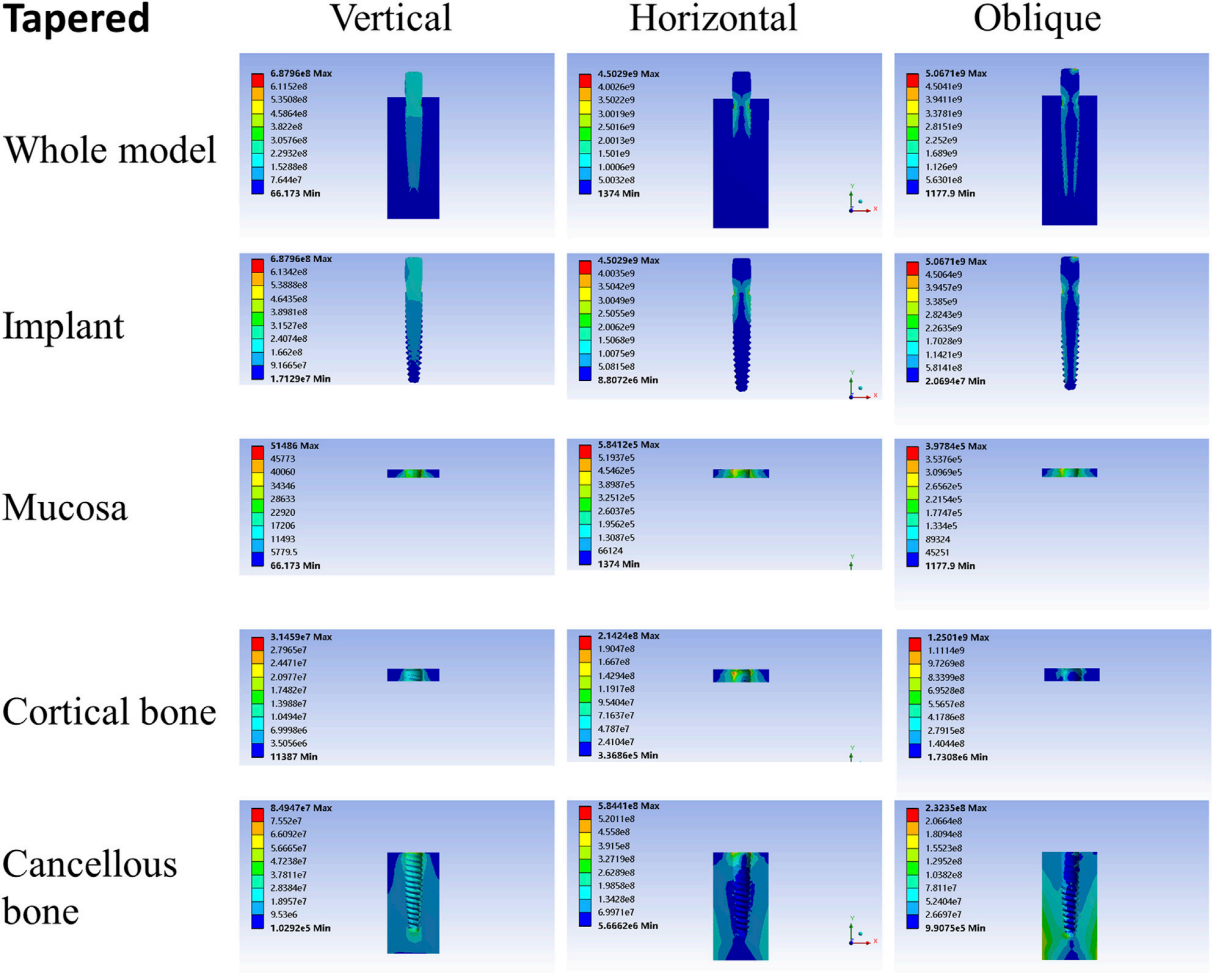
Stress distribution of the tapered implant under 100-N load.

For different load directions, when a vertical load was applied to the models, the maximum stresses in the implants and surrounding tissues, regardless of the root-analog or taper implants, were lowest, and the stress was balanced distributed in the whole model compared with a horizontal and oblique load. In contrast, the stress in each implant was concentrated on its cervix relatively; stress in the mucosa and cortical bone was distributed around the cervix of the implants, with maximum stress occurring near the implants (Tables 2–4). For the cancellous bone, stress was mainly concentrated at the root tip (Figures 2–8). When the load was applied horizontally, the maximum stress in the models was the highest among the three kinds of loads, and the stress distribution was highly concentrated on the specific areas. The stress in each implant was concentrated on its cervix and on the opposite side of the loading point, the lingual side. Likewise, the stress in the mucosa and cortical bone were both concentrated on the lingual side and near the junction of the implant. In the cancellous bone, the stress was concentrated in two locations: the first location was on the same side of the loading point where the implant apex contacted the cancellous bone, whereas the second location was near the implant cervix, which was under the junction of the implant and cortical bone, and was on the opposite side of loading point (Figures 2–8). When an oblique load was applied, the maximum stress in the implants and surrounding tissues was between those observed from the vertical and the horizontal load (Tables 2–4). The stress distribution was similar to the horizontal load. The stress concentration positions in the implants, mucosa, and cortical bone were the same under the horizontal load. However, the stress in the cancellous bone was concentrated in two locations near the concentrated area under the horizontal load. The oblique stress concentration area on the same side was a little bit upon the horizontal stress concentration area and on the opposite side. The stress concentration area shifted below the horizontal stress concentration area (Figures 2–8).

**TABLE 2:** Maximum stress values when RAIs and tapered implant are subjected to 100-N vertical load.

Dental implant models	Implant (MPa)	Mucous membrane (MPa)	Cancellous bone (MPa)	Cortical bone (MPa)
26	5.4983	0.0019240	2.0921	4.2940
46	6.1727	0.0021796	1.6009	4.9725
34	14.5890	0.0053000	3.5547	12.7400
13	12.7000	0.0041022	2.3722	6.6353
12	17.2340	0.0030784	3.6631	10.702
11	13.2870	0.0030641	2.0395	9.3141
Tapered implant	67.4090	0.0059824	6.6965	6.1804

**TABLE 3:** Maximum stress values when RAIs and tapered implant are subjected to 100-N horizontal load.

Dental implant models	Implant (MPa)	Mucous membrane (MPa)	Cancellous bone (MPa)	Cortical bone (MPa)
26	14.5640	0.0049187	2.7979	11.2400
46	27.8630	0.0092178	2.6464	22.9080
34	53.4920	0.0185430	6.5350	46.7240
13	16.9160	0.0096245	5.1270	17.9760
12	49.5080	0.0154300	14.7280	43.431
11	21.6190	0.0082758	2.6395	29.1180
Tapered implant	578.9900	0.0912870	32.0940	64.8470

**TABLE 4:** Maximum stress values when RAIs and tapered implant are subjected to 100-N oblique load.

Dental implant models	Implant (MPa)	Mucous membrane (MPa)	Cancellous bone (MPa)	Cortical bone (MPa)
26	20.3480	0.0074868	3.5900	16.5680
46	22.3470	0.0077840	2.8923	19.6660
34	42.8330	0.0151570	5.8313	37.8790
13	9.8724	0.0065159	3.8088	12.1410
12	31.7050	0.0078445	7.7121	30.190
11	12.0520	0.0034788	2.9090	12.1230
Tapered implant	341.0300	0.0496360	19.9350	37.8830

As far as the root number was concerned, in group A, the maximum stress in the multiple-root implant (Figures 2, 3) and the surrounding tissues was significantly less than that in a single root implant, including the taper implant (Figures 4–8), under all the loading directions. At the same time, the difference of maximum stress between the implants with three roots and two roots was not considerable under either the vertical or oblique load. However, the maximum stress in the double-root implants under horizontal load was more significant than that in the triple-root implant (Figures 2, 3). On the other hand, when the root number was the same and the length was different, in the canine model, which had a single but longer root in group B, the maximum stress in the implant and cancellous bone were lesser than that in the incisor model. Inversely, the maximum stresses in the mucosa and cortical bone were higher than in the incisor (Figures 5, 7).

When the root number and length were similar, like group C, the length–width ratio of the root cross-section affected the stress distribution. Compared with the mandibular first premolar, the root cross-section of the maxillary central incisor was more rounded. When the vertical load was applied on both models, the stress distribution was parallel. However, when subjected to the horizontal and oblique loads, the results in the mandibular first premolar and its surrounding tissues were more significant than that in the maxillary central incisor, while the stress concentration areas were located in the same position (Figures 4, 7).

As shown in Tables 2–4, the taper implant’s FEA results were considerably different from that of the root-analog implants, like their morphological difference. Under the vertical load, the stress in the taper implant model was more concentrated in the implant itself. The maximum stress in the mucosa and cortical bone was similar to the single-root teeth models. However, the maximum stress in cancellous bone was greater than in the root-analog implants model, especially under the horizontal and oblique loads (Tables 3, 4). When subjected to horizontal and oblique load, the only similar maximum stress in the mucosa and cortical bone among the taper implant and lateral incisor as described before dismissed, the maximum stress in all surrounding tissues, let alone in the implants, were several or even dozens of times greater than that in the root-analog implant models. For example, the maximum stress in the taper implant was about 40 times greater than that in the maxillary first molar implant, while the maximum stress in the mucosa was 18 times. The maximum stresses in the taper implant under the oblique load were approximately between the vertical and horizontal loads. Similar to the results of the horizontal load, the maximum stress in the implant itself was dozens of times greater than that in the root-analog implants when subjected to 45° load. Overall, under all loads from three directions, the maximum stress in tissues surrounding the taper implants was generally greater than that in the anatomical-shaped implant models. Although, the taper implant itself has taken most of the stress, its surrounding tissue is still subjected to greater stress.

## Discussion

The most commonly used dental implants in clinical practice are taper in shape. Watanabe et al., conducted FEA of the tapered implants, and their results are consistent with the present study (Watanabe et al., 2003; He et al., 2017; Dantas et al., 2020; Oliveira et al., 2020). In FEA, the vertical load simulates the stress distribution in teeth or implants during cutting and chewing food. The horizontal and oblique loads trigger the functional movement of grinding and chewing. When the loading value remains the same, maximum stress can reflect the stress concentration level, excluding the periodontal membrane cushion. As for the same load from different directions, the vertical load, which is parallel to the long axis of the implants, results in minor stress. The horizontal load leads to the highest in all implant models.

On the other hand, the stress on the taper implant and surrounding tissues, regardless of loading direction, was significantly greater than that on every root-analog implant under the same load in most cases. Moreover, the stress distribution in the root-analog implants was generally equalized, while the taper implant was concentrated in a specific area. The maxillary lateral incisor and mandibular first premolar were exceptions, the stress distribution of which was similar to the taper implant, probably because of the similitude of their geometry.

The geometry of teeth or implants is of great significance to disperse masticatory stress. The stress concentration in the taper implant may increase the risk of damage to the implant and surrounding tissues, such as bone resorption and peri-implantitis, and consequently, cause implant failure (Duyck et al., 2001; Gotfredsen et al., 2002; Bing et al., 2020). In contrast, root-analog implants, especially multiple-root implants and implants with longer roots, can disperse the same load through their natural geometry, thus reducing the stress in the taper implant. This stress distribution protects the root-analog implant from stress concentration during daily mastication (He et al., 2017; Dantas et al., 2020).

Even if the implant’s shape remains taper, the thread’s diameter, length, and modification are also responsible for different stress distribution (Kong et al., 2008; Eraslan and İnan, 2010; Yamanishi et al., 2014). The increase in diameter and length could more precisely distribute stress. Thus, it appears logical that the root-analog implants from different neutral teeth show different stress distributions. The first maxillary molar can disperse the stress more effectively than any other root-analog implant, seemingly due to the number and shape of its root. The relationship between the implant geometry, including root length, root number, and the shape of the cervical cross-section and their corresponding function, has been discussed in the following paragraphs.

Among the implant geometry, root length significantly impacts the stress distribution. The maxillary cuspid is much longer than the maxillary lateral incisor. Furthermore, both have a single root and a similar length–width ratio of the root cross-section. However, the maximum stresses in the cuspid was lower than that in lateral incisor under the vertical load. As the stress concentration area in the lateral incisor can be observed in the root cervix and apex, such area occurred at the apex of the cuspid only, and its root cervix did not suffer from stress concentration. Additionally, in the FEA model of the lateral incisor, the stress in the mucosa and cortical bone was similar to that in the cuspid, but the stress in the cancellous bone was much greater than that in the cuspid. The longer the root stress is presumably distributed along its root and cancellous bone instead of being concentrated on the cervix, which was observed in the taper implants with different lengths (Ding et al., 2009; Pirmoradian et al., 2020). Thus, a taper implant with the same length is suitable for restoring the missing tooth. Since single-root teeth, including incisor and canine, are involved in cutting and penetrating food and supporting facial contours, they mainly use small and vertical force (Po et al., 2011).

Nevertheless, when the single-root implants and the taper implant are subjected to horizontal load, the stress concentration appears in the specific area. Under the horizontal load, the maximum stress in the taper implant and its surrounding tissues were about 40 times greater than that in the maxillary first molar model and 20 times greater than that in the first mandibular molar model. This drawback could contribute to the damage to the cortical bone and mucosa around the implant cervix. As the maxillary first molar has three roots forming a tripod-like structure, it is comparatively stable when subjected to either vertical or horizontal load, so does the first mandibular molar. Because they could effectively disperse the stress to each root, the tooth with more roots has less stress as each root partakes under the same load. This effect enables the molars to bear the level component of force from the masticatory movement without the formation of stress concentration.

The rounded root cross-section, or namely more taper root, may be conducive to stress distribution when comparing the maxillary central incisor and mandibular first premolar. But in this case, the taper implant was supposed to show a more uniform stress distribution, contrary to our results. The presence of thread on the taper implant can be one of the possible reasons. However, further investigations are needed to understand this phenomenon more precisely.

In summary, when subjected to the same magnitude and direction of load mimicking masticatory force, the stress distribution in titanium root-analog implants and surrounding tissues was significantly reduced than that in the taper implant. Because of the differences in implant length, root shape, and the number of roots, the root-analog implants can disperse the stress uniformly and be less susceptible to stress concentration. Hence, diversity in implant shape is required, so that the strength and direction of the load can vary on different teeth, and root-analog implant could be an alternative. Furthermore, we will investigate to verify its feasibility *in vivo* and probe its connection with crowns.

## Data Availability

The original contributions presented in the study are included in the article/Supplementary Material. further inquiries can be directed to the corresponding authors.
