# Supplementary material for: Biomechanical Comparison of Six Different Root-Analog Implants and the Conventional Morse Taper Implant by Finite Element Analysis
Source: Front Genet. 2022 Jun 13;13:915679. doi: 10.3389/fgene.2022.915679 (PMC9234945; doi:10.3389/fgene.2022.915679)
Supplement: Supplementary file 1 [file DataSheet1.docx]

**Table 1.** Elastic modulus and Poisson ratio of different materials.

| **Material** | **Elastic Modulus (GPa)** | **Poisson Ratio (μ)** |
| --- | --- | --- |
| Cortical bone | 13.7 | 0.30 |
| Cancellous bone | 1.85 | 0.30 |
| Mucosa | 0.003 | 0.45 |
| Titanium | 110 | 0.35 |

Table 2. Maximum stress values when RAIs and tapered implant are subjected to 100 N vertical load.

| **Dental implant models** | **Implant (MPa)** | **Mucous membrane (MPa)** | **Cancellous bone (MPa)** | **Cortical bone (MPa)** |
| --- | --- | --- | --- | --- |
|  |  |  |  |  |
| 26 | 5.4983 | 0.0019240 | 2.0921 | 4.2940 |
| 46 | 6.1727 | 0.0021796 | 1.6009 | 4.9725 |
| 34 | 14.5890 | 0.0053000 | 3.5547 | 12.7400 |
| 13 | 12.7000 | 0.0041022 | 2.3722 | 6.6353 |
| 12 | 17.2340 | 0.0030784 | 3.6631 | 10.702 |
| 11 | 13.2870 | 0.0030641 | 2.0395 | 9.3141 |
| Tapered implant | 67.4090 | 0.0059824 | 6.6965 | 6.1804 |

Table 3. Maximum stress values when RAIs and tapered implant are subjected to 100 N horizontal load.

| **Dental implant models** | **Implant (MPa)** | **Mucous membrane (MPa)** | **Cancellous bone (MPa)** | **Cortical bone (MPa)** | |
| --- | --- | --- | --- | --- | --- |
|  |  |  |  | |  |
| 26 | 14.5640 | 0.0049187 | 2.7979 | | 11.2400 |
| 46 | 27.8630 | 0.0092178 | 2.6464 | | 22.9080 |
| 34 | 53.4920 | 0.0185430 | 6.5350 | | 46.7240 |
| 13 | 16.9160 | 0.0096245 | 5.1270 | | 17.9760 |
| 12 | 49.5080 | 0.0154300 | 14.7280 | | 43.431 |
| 11 | 21.6190 | 0.0082758 | 2.6395 | | 29.1180 |
| Tapered implant | 578.9900 | 0.0912870 | 32.0940 | | 64.8470 |

Table 4. Maximum stress values when RAIs and tapered implant are subjected to 100 N oblique load.

| **Dental implant models** | **Implant (MPa)** | **Mucous membrane (MPa)** | **Cancellous bone (MPa)** | **Cortical bone (MPa)** |
| --- | --- | --- | --- | --- |
|  |  |  |  |  |
| 26 | 20.3480 | 0.0074868 | 3.5900 | 16.5680 |
| 46 | 22.3470 | 0.0077840 | 2.8923 | 19.6660 |
| 34 | 42.8330 | 0.0151570 | 5.8313 | 37.8790 |
| 13 | 9.8724 | 0.0065159 | 3.8088 | 12.1410 |
| 12 | 31.7050 | 0.0078445 | 7.7121 | 30.190 |
| 11 | 12.0520 | 0.0034788 | 2.9090 | 12.1230 |
| Tapered implant | 341.0300 | 0.0496360 | 19.9350 | 37.8830 |
